# Sphingomonas paucimobilis Bacteremia in a Preterm Neonate: A Rare Cause of Early-Onset Sepsis

**DOI:** 10.7759/cureus.110154

**Published:** 2026-06-02

**Authors:** Malini Jagannatha Rao, Karthik Bhaskaran, Mohini Singh

**Affiliations:** 1 Department of Microbiology, ESIC Medical College, PGIMSR and Model Hospital, Bengaluru, IND; 2 Department of Microbiology, PESU Institute of Medical Sciences and Research, Bengaluru, IND

**Keywords:** antimicrobial therapy, neonatal sepsis, preterm neonate, rare pathogen, sphingomonas paucimobilis, vitek 2

## Abstract

*Sphingomonas paucimobilis (S. paucimobilis)*, an environmental Gram-negative bacillus, is a rare cause of clinical infections in neonates. This case report describes a preterm neonate with early-onset sepsis (EOS) caused by *S. paucimobilis*. Identification was achieved using the VITEK 2 system, and targeted antimicrobial therapy with meropenem, vancomycin, and metronidazole resulted in clinical improvement. Early recognition and microbiological diagnosis are essential for managing unusual pathogens in neonatal intensive care settings.

## Introduction

Neonatal sepsis is a critical clinical condition associated with high morbidity and mortality, particularly in preterm infants. Early-onset sepsis (EOS), occurring within the first 72 hours of life, is commonly caused by vertical transmission of pathogens. While Gram-negative organisms such as *Escherichia coli (E. coli)* and *Klebsiella* spp. are typical culprits, rare environmental bacteria like *Sphingomonas paucimobilis (S. paucimobilis)* are emerging as causes [1,2,3]. S. paucimobilis is a non-fermenting, yellow-pigmented, aerobic Gram-negative bacillus usually found in soil and hospital water systems [1,4]. It has been recognized as a low-virulence organism; however, in vulnerable populations such as neonates, it can cause severe bloodstream infections [2,3,5,6]. We present a case of EOS caused by *S. paucimobilis* in a preterm neonate, diagnosed by automated identification and successfully managed with culture-guided antibiotics.

## Case presentation

The patient was a female preterm neonate, born at 34 weeks and six days of gestation to a G3P1L1A1 mother and delivered in transit on April 2, 2025. The baby cried immediately after birth and weighed 1.86 kg at admission. She was admitted with respiratory distress and signs of compensated shock, with a Grading of Acute Distress in Sick Newborns (GADS) score of 4/10. Early-onset neonatal sepsis, defined as sepsis occurring within the first 72 hours of life and usually resulting from vertical transmission of organisms from the mother to the infant during labor or delivery, was clinically suspected. Initial vital parameters revealed respiratory distress requiring continuous positive airway pressure (CPAP) support, with features of poor perfusion and tachycardia consistent with compensated shock. Empirical antibiotic therapy was initiated with intravenous cefotaxime and amikacin, in accordance with standard neonatal sepsis protocols, along with supportive care.

By day three of life (DOL 3), the neonate showed partial clinical stabilization, with improved respiratory effort and hemodynamics. However, on DOL 4, the infant developed multiple episodes of vomiting with brownish gastric aspirates, suggestive of gastrointestinal intolerance or an evolving systemic illness. The clinical condition deteriorated on DOL 5, when the baby became lethargic, with poor perfusion and bradycardia (heart rate <100/min), consistent with progression to septic shock. Fresh frozen plasma was transfused as part of the supportive management.

In view of clinical deterioration, antibiotic therapy was escalated to intravenous piperacillin-tazobactam. A blood culture obtained during this period became positive after 48 hours of incubation in the BacT/ALERT system (bioMérieux, Marcy-l’Étoile, France). Subculture on blood agar and MacConkey agar, followed by identification and antimicrobial susceptibility testing using the VITEK 2 Compact system (bioMérieux), confirmed the isolate as *S. paucimobilis*, an aerobic, non-fermenting, Gram-negative bacillus associated with healthcare-associated and opportunistic infections [1,2]. 

Antimicrobial susceptibility testing showed resistance to third-generation cephalosporins and susceptibility to meropenem and aminoglycosides. Based on culture results and clinical status, therapy was modified to intravenous meropenem (37.2 mg every eight hours). Vancomycin was continued as part of empirical Gram-positive coverage in neonatal sepsis until full clinical stabilization, despite having no activity against Gram-negative organisms. Metronidazole (14 mg daily) was added empirically to cover possible anaerobic gastrointestinal infection in view of persistent vomiting and gastric aspirates, although anaerobic infection was not confirmed microbiologically.

Following targeted therapy and supportive management, the neonate showed progressive clinical improvement. Vomiting resolved, enteral feeds were gradually reintroduced, and respiratory and hemodynamic parameters remained stable. The infant was successfully weaned off CPAP and inotropic support and was discharged in stable condition.

Materials and methods

Blood was collected under aseptic precautions and inoculated into aerobic bottles of the BacT/ALERT 3D system (bioMérieux). Upon flagging positive, subcultures were done on blood agar and MacConkey agar and incubated at 37 °C for 18-24 hours. Organism identification and AST were performed using the VITEK 2 Compact System with GN ID and AST cards (bioMérieux). Interpretation of susceptibility results followed Clinical and Laboratory Standards Institute (CLSI) 2023 guidelines [7]. *Escherichia coli* ATCC 25922 and *Pseudomonas aeruginosa* ATCC 27853 were used as quality control strains.

## Discussion

*S. paucimobilis* is an aerobic, non-fermenting, Gram-negative bacillus commonly found in soil, water, and hospital environments, including water pipelines and medical devices [1,4,8]. Although it is considered a low-virulence organism due to the absence of lipopolysaccharide endotoxin in its outer membrane, it has increasingly been reported as a cause of healthcare-associated infections, particularly in immunocompromised patients and neonates [1,2,3]. Neonatal infections due to *S. paucimobilis* are rare but clinically important. Isolation of this organism from sterile sites such as blood should not be dismissed as contamination, especially when accompanied by clinical features consistent with sepsis. In the present case, the organism was isolated from a blood culture in a preterm neonate with progressive clinical deterioration, supporting its pathogenic role.

The infant initially received empirical therapy with cefotaxime and amikacin, which did not result in sustained clinical improvement. This finding is consistent with previous reports describing reduced susceptibility of *S. paucimobilis* to third-generation cephalosporins and some beta-lactams, likely due to chromosomally encoded beta-lactamases [1,3]. Blood culture identification using the VITEK 2 Compact system allowed timely recognition of the organism and determination of its antimicrobial susceptibility. It is important to note that vancomycin was used as part of empirical neonatal sepsis coverage to address potential Gram-positive pathogens, particularly during the phase of septic shock. However, vancomycin has no activity against Gram-negative organisms such as *S. paucimobilis* and was not targeted toward this isolate. Similarly, metronidazole was administered empirically due to gastrointestinal symptoms, although no anaerobic pathogens were isolated.

The clinical course demonstrates the importance of considering atypical environmental organisms in neonatal sepsis, particularly when there is an inadequate response to standard empirical therapy. It also highlights the utility of automated blood culture and identification systems in enabling early pathogen detection and optimization of antimicrobial therapy. During the episode of clinical deterioration, inflammatory markers, including C-reactive protein (CRP), procalcitonin, erythrocyte sedimentation rate (ESR), and serum lactate, showed a pattern consistent with severe bacterial sepsis, with elevated CRP and procalcitonin levels and raised serum lactate reflecting tissue hypoperfusion. Nevertheless, the temporal association between clinical worsening, blood culture positivity for *S. paucimobilis*, and subsequent clinical improvement following targeted antimicrobial therapy strongly supports the pathogenic significance of the isolate. Overall, this case underscores the need for vigilance toward uncommon opportunistic pathogens in neonatal ICUs and reinforces the importance of microbiological confirmation to guide rational, evidence-based antimicrobial therapy [8-13].

## Conclusions

*S. paucimobilis* should be considered a rare but significant cause of neonatal sepsis, especially in preterm infants presenting with clinical deterioration despite empirical antibiotic therapy. Early microbiological identification and antimicrobial susceptibility testing are essential for the timely initiation of targeted antimicrobial therapy. In our case, prompt modification of antibiotics based on culture results led to significant clinical improvement and recovery of the patient. Increased awareness of this emerging opportunistic pathogen may help improve diagnostic accuracy and patient outcomes in neonatal intensive care settings.
